# Comparison of external dose estimates using different retrospective dosimetry methods in the settlements located near Semipalatinsk Nuclear Test Site, Republic of Kazakhstan

**DOI:** 10.1093/jrr/rrad082

**Published:** 2023-11-18

**Authors:** Valeriy Stepanenko, Sergey Shinkarev, Andrey Kaprin, Kazbek Apsalikov, Sergey Ivanov, Peter Shegay, Evgenia Ostroumova, Ausrele Kesminiene, Alexandra Lipikhina, Viktoria Bogacheva, Kassym Zhumadilov, Masayoshi Yamamoto, Aya Sakaguchi, Satoru Endo, Nariaki Fujimoto, Bernd Grosche, Vladimir Iatsenko, Alla Androsova, Zukhra Apsalikova, Noriyuki Kawano, Masaharu Hoshi

**Affiliations:** A. Tsyb Medical Radiological Research Centre - Branch of the National Medical Research Radiological Centre of the Ministry of Health of the Russian Federation, 4 Koroleva St., Obninsk, Kaluga Region, 2490036, Russian Federation; State Research Center - Burnasyan Federal Medical Biophysical Center of Federal Medical Biological Agency, 46 Zhivopisnaya St., Moscow, 123098, Russian Federation; National Medical Research Radiological Centre of the Ministry of Health of the Russian Federation, 4 Koroleva St., Obninsk, Kaluga Region, 2490036, Russian Federation; Peoples' Friendship University of Russia, 6 Miklukho-Maklaya St., Moscow, 117198, Russian Federation; P.A. Hertzen Moscow Oncology Research Institute-branch of the National Medical Research Radiological Centre of the Ministry of Health of the Russian Federation, 2nd Botkinsky Drive 3, Moscow, 125284, Russian Federation; Scientific Research Institute of Radiation Medicine and Ecology of the non-commercial joint-stock company «Semey Medical University», 258 Gagarin St., Semey, 071407, Republic of Kazakhstan; A. Tsyb Medical Radiological Research Centre - Branch of the National Medical Research Radiological Centre of the Ministry of Health of the Russian Federation, 4 Koroleva St., Obninsk, Kaluga Region, 2490036, Russian Federation; Peoples' Friendship University of Russia, 6 Miklukho-Maklaya St., Moscow, 117198, Russian Federation; National Medical Research Radiological Centre of the Ministry of Health of the Russian Federation, 4 Koroleva St., Obninsk, Kaluga Region, 2490036, Russian Federation; Environment and Lifestyle Epidemiology Branch, International Agency for Research on Cancer/WHO, 25 avenue Tony Garnier, Lyon, 69366, France; Environment and Lifestyle Epidemiology Branch, International Agency for Research on Cancer/WHO, 25 avenue Tony Garnier, Lyon, 69366, France; Scientific Research Institute of Radiation Medicine and Ecology of the non-commercial joint-stock company «Semey Medical University», 258 Gagarin St., Semey, 071407, Republic of Kazakhstan; A. Tsyb Medical Radiological Research Centre - Branch of the National Medical Research Radiological Centre of the Ministry of Health of the Russian Federation, 4 Koroleva St., Obninsk, Kaluga Region, 2490036, Russian Federation; L.N. Gumilyov Eurasian National University, 13 Munaitpasova St., office 300, Astana, 010008, Republic of Kazakhstan; Low-Level Radioactivity Laboratory, Institute of Nature and Environmental Technology, Kanazawa University, Wakemachi O24, Nomi, Ishikawa, 923-1224, Japan; Institute of Pure and Applied Sciences, University of Tsukuba 1-1-1 Tennodai, Tsukuba, Ibaraki, 305-8577, Japan; Graduate School of Advanced Science and Engineering, Hiroshima University 1-4-1, Kagamiyama, Higashi, Hiroshima, 739-8527, Japan; Research Institute for Radiation Biology and Medicine, 1-2-3, Kasumi, Minami-ku, Hiroshima, 734-8553, Japan; Consultant, formerly: Federal Office for Radiation Protection, Germany, Grasmueckenweg 19, 85356 Freising, Germany; State Research Center - Burnasyan Federal Medical Biophysical Center of Federal Medical Biological Agency, 46 Zhivopisnaya St., Moscow, 123098, Russian Federation; State Research Center - Burnasyan Federal Medical Biophysical Center of Federal Medical Biological Agency, 46 Zhivopisnaya St., Moscow, 123098, Russian Federation; Scientific Research Institute of Radiation Medicine and Ecology of the non-commercial joint-stock company «Semey Medical University», 258 Gagarin St., Semey, 071407, Republic of Kazakhstan; The Center for Peace, Hiroshima University Higashisenda-machi 1-1-89, Naka-ku, Hiroshima, 730-0053, Japan; The Center for Peace, Hiroshima University Higashisenda-machi 1-1-89, Naka-ku, Hiroshima, 730-0053, Japan

**Keywords:** radioactive fallout, nuclear weapon tests, retrospective dosimetry, external dose, population exposure

## Abstract

For correct assessment of health risks after low-dose irradiation, calculation of radiation exposure estimates is crucial. To verify the calculated absorbed doses, instrumental methods of retrospective dosimetry are used. We compared calculated and instrumental-based estimates of external absorbed doses in the residents of Dolon, Mostik and Cheremushki villages, Kazakhstan, affected by the first nuclear weapon test performed at the Semipalatinsk Nuclear Test Site (SNTS) on August 29, 1949. The ‘instrumental’ doses were retrospectively estimated using the Luminescence Retrospective Dosimetry (LRD) and Electron Spin Resonance (ESR) methods. Correlation between the calculated individual cumulative external absorbed whole-body doses based on typical input data and ESR-based individual doses in the same people was strong (r = 0.782). It was even stronger between the calculated doses based on individual questionnaires’ input data and the ESR-based doses (r = 0.940). Application of the LRD method is useful for validation of the calculated settlement-average cumulated external absorbed dose to air. Reconstruction of external exposure can be supplemented with the data from later measurements of soil contamination with long-lived radionuclides, such as, ^137^Cs. Our results show the reliability of the calculational method used for the retrospective assessment of individual external doses.

## INTRODUCTION

The first nuclear test in the USSR was conducted at the Semipalatinsk Nuclear Test Site (SNTS) on August 29, 1949 [1]. During the following 40 years, 456 nuclear explosions were carried out, including 111 atmospheric tests (86 events in air and 25 surface events) between 1949 and 1963. After the onset of the Limited Test Ban Treaty signed in 1963, the tests at the SNTS were restricted to underground. The last test was conducted at the SNTS on October 19, 1989 [1, 2]. The atmospheric tests largely contributed to the radioactive contamination of the environment and to the radiation exposure of the population [2]. The total yield of these events was 6.58 Mt. The SNTS was closed on August, 1991, and the National Nuclear Center of the Republic of Kazakhstan is located on this territory.

Radiation dose assessment in the residents of settlements near the former SNTS during the testing period is important for correct epidemiological calculation of risks of low-dose radiation health effects [3–5]. Various retrospective dosimetry methods are used for dose estimation in case of uncontrolled radiation exposure that took place in the past during nuclear testing [6–8]. Mainly, these methods are computational. It should be noted that any calculated dose estimation has an essential potential uncertainty due to a possible lack of initial information that is needed for retrospective dose estimation many years after the uncontrolled irradiation. Therefore, it is not surprising that some of these estimates are conflicting and a subject of international discussions [6, 7]. The use of instrumental methods of retrospective dosimetry to verify the calculated doses is very important to ensure that the calculated estimates are realistic. The instrumental methods of retrospective dosimetry include Luminescence Retrospective Dosimetry (LRD) method by quartz inclusions in man-made objects of the environment (e.g. bricks of buildings ‘witnessed’ radiation exposure) [3, 9–17] and Electron Spin Resonance (ESR) method [18–22] by enamel samples of teeth extracted from people who lived near the nuclear test site during the testing period. Reconstruction of radiation doses using the results of measurements of long-lived radionuclides in soil may also be useful [23, 24]. Doses from internal irradiation, which can only be estimated via calculation, are expected to be much less than external irradiation doses in case of nuclear tests (with exception of thyroid internal exposure to iodine radionuclides) [25, 26].

Methodology of retrospective external dose calculations has been recently published [27–30]. This methodology is based on available archival data on dose rate to air measured near the study settlements.

Data obtained by the LRD and ESR methods with available quartz containing bricks and teeth enamel samples were used for dose estimates comparison [8–11, 13–22]. The dose reconstruction approaches based on contemporary measurements of long-lived radionuclides in soil of the study settlements [23, 24] were used as well.

The article is focused on the comparison between external dose estimates based on calculations and instrumental methods of retrospective dosimetry for several settlements located near the SNTS. The comparison was also made for selected individuals, who were residents of these settlements, and for whom the individual information on residence history and lifestyle was available.

## MATERIALS AND METHODS

### Study settlements

**Fig. 1:**
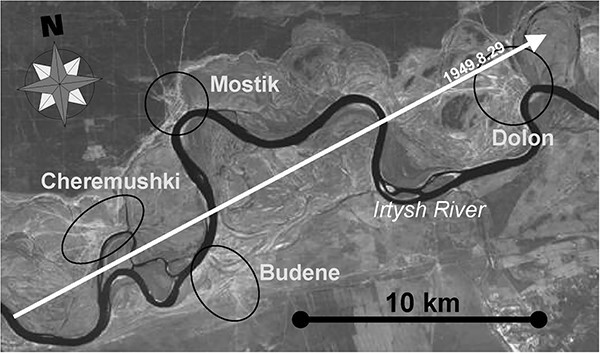
Location of the radioactive plume formed after the first SNTS nuclear test on August 29, 1949 [31].

Three villages, Dolon, Mostik, and Cheremushki, Kazakhstan, were selected for comparison of external doses estimated using different retrospective dosimetry methods. Radioactive contamination in these villages resulted from the first nuclear weapon test at the SNTS on August 29, 1949, and these settlements are located just near the centerline of radioactive trace (Fig. 1).

The selection was based on the availability of data on radiological conditions in these villages, such as dose rates at the time of radioactive fallout, ^137^Cs soil contamination density [1, 6, 27, 31–39]. Using these data, it is possible to calculate the average external dose to air in these settlements. The majority (about 95–99%) of the whole lifetime external dose was received by the residents of the contaminated villages during the first year after the test [6, 24, 27]. Therefore, for retrospective dose calculations it is sufficient to consider input parameters for a year from August 29, 1949, and persons who resided in the study villages at the time of the first SNTS nuclear test and one year after.

### Methodologies of retrospective estimation of external dose to air

#### Methodology of calculation of external dose to air

Studies describe the approaches of retrospective external dose computation [27, 28, 33] and this can also be seen in more recent studies [29, 30]. Retrospective calculations of external radiation dose after a nuclear explosion are products of two components. The first component relates to the calculation of absorbed dose to air at the location where the corresponding measurements of absorbed dose rate after the explosion were carried out. The second component relates to calculation of the individual absorbed external exposure dose to residents of a study settlement. The latter is described below.

The following main parameters should be considered when calculating absorbed dose to air [27–30, 33]. They include date, time and location of the test; composition of fission material; total yield of the explosion; the height of detonation above ground surface; the height of the radioactive cloud top; wind speed, averaged up to the height of radioactive cloud; distance from the location of explosion to a study settlement (Table 1).

**Table 1:** Main parameters related to the first SNTS nuclear test on August 29, 1949

Parameter	Value [Reference]
Date of explosion	29.08.1949 [1, 2, 35]
Time of explosion (local)	7:00 [37]
Location	Location “Π-1”, SNTS [37]
Composition of fission material	Pu-239 [35, 37]
Total yield	22 kt [1, 2, 35]
Height above ground	30 m (tower) [35, 37]
Height of radioactive cloud top	9 km [32, 37]
Average wind speed	47–60 km/h [32, 37]
Distance from the location of explosion to the settlement:	
Dolon	118 km [35]
Mostik	90 km [35]
Cheremushki	76 km [35]

We chose the joint USA/Russian methodology for external dose calculation after the first SNTS test on August 29, 1949. [27, 28] In this approach, the time dependence of exposure-rate to air was derived from the U.S. nuclear test data (TRINITY test) [29], which was very similar to the first SNTS test. Dose rates along the radioactive trace in the study settlements were taken from the available results of ground and aerial exposure rate measurements during the period from September 5 to September 13, 1949, after the first SNTS test [33–37]. To convert historical exposure rate units (mR/hour) to absorbed dose rate to air (mGy/hour), a conversion factor 8.7 × 10^−3^ mGy/mR was used.

As far as the measured dose rates near Dolon village mainly refer to the radioactive trace, and not to the settlement itself [34], an accumulated dose to air in Dolon was calculated in accordance with the available information about dose-rate gradient near the village [37], and radioactive contamination in the direction from the radioactive trace into the settlement [12] which is close to the radioactive trace (Fig. 1).

#### Luminescence retrospective dosimetry methodology

Studies describe the LRD dose reconstruction methodology with quartz containing brick samples collected in the radioactively contaminated settlements[8–10, 13]. This methodology is based on the use of measurements of the intensity of thermoluminescence (TL) or optically stimulated luminescence (OSL) in the quartz microcrystals extracted from the buildings’ bricks sampled in territories near to SNTS. The quartz grains are considered as natural luminescent dosimeters. The automatic reader TL/OSL-DA-15 (RISO, Denmark) was used for measurements of stimulated luminescence intensity. The calibration dose dependencies of extracted quartz grains were performed using built-in ^90^Sr/^90^Y beta source that is calibrated against a secondary standard ^137^Cs photon source.

LRD techniques were applied to quartz grains extracted from each brick to determine the total cumulative absorbed dose, D_tot_. The total cumulative absorbed dose is the sum of dose contributions from artificial (accidental) and natural (background, D_bg_) ionizing radiation exposure. The presence and value of artificial irradiation was determined by subtraction D_bg_ from D_tot_. Assessment of D_bg_ value is based on the ‘age’ of building and, assessment of concentrations of natural radionuclides within bricks and surrounding environment, and estimated dose rates. Another, more direct possibility to estimate D_bg_ is based on luminescence measurements of quartz grains extracted from the bricks sampled inside the well shielded parts of considered buildings. The difference between D_tot_ and D_bg_ is converted to dose to air at a reference location, to allow comparisons with estimates of cumulative dose from model-based calculations. The conversion factor, defined as an inverse of a ratio of absorbed dose in brick to air dose at the reference location, usually calculating for a given energies of gamma-irradiation and geometries based on Monte-Carlo (MC) simulations. [9]

#### Methodology of external dose reconstruction using measurements of radionuclides contamination in soil

See [23] for the approach for external dose reconstruction using the results of measurement of long-lived radionuclides in soil. There is another approach for external dose reconstruction based on the results of contemporary measurements of soil contamination with ^137^Cs in the village of Dolon used by Imanaka *et al.* [24]. Applying this methodology, external accumulated doses for Dolon village was estimated using available ^137^Cs soil contamination data and calculating the input to the dose from gamma-emitting radionuclides from ^239^Pu fission considering temporal change in the fission product composition [24]. Twenty-nine radionuclides from the list of fission products were selected [24], as possible candidates that could significantly contribute to the external accumulated dose from the first SNTS test. Rare gas species and short half-life radionuclides were excluded considering the fallout arrival time of ~1,5–3 h in the study settlements. Neutron-capture nuclides were not considered because their input to the annual value of external dose is much less compared with the fission products [24, 33].

Shortly, the approach, described in detail in [24], was the following.

1) The fission products nuclear data were used as a basis to calculate temporal change of fission products composition after the nuclear explosion.

2) Dose rate conversion coefficients from a unit density on the ground to the absorbed dose to air at 1 m above the ground were calculated for each considered radionuclide using published gamma-ray emission data.

3) Absorbed dose rate to air at 1 m above the ground at time t after the explosion was expressed in the form of equation accounting for:

-contemporary measurements of ^137^Cs soil contamination density (time dependence from initial activity at the moment of deposition according to ^137^Cs decay constant);

-activity ratio at time t of each considered radionuclide to ^137^Cs activity in a mixture maintaining the same composition as fission products;

-fractionation factor for each considered radionuclide, indicating divergence of fallout composition from fission products composition at the time of deposition;

-dose rate conversion coefficient per unit of soil contamination density for each considered radionuclide.

As a result, a sum of products of each factor listed above under the item 3 provides a time-dependent dose rate to air at 1 m above the ground. The cumulative dose to air was obtained by integrating this dose rate for the period starting from the time of deposition.

The data on ^137^Cs soil contamination density [31, 38, 39] were used for retrospective external dose estimations for the study villages.

Based on the previous experience in soil sampling for radionuclide measurements for retrospective dosimetry purpose after the Chernobyl accident and in the areas around the SNTS [39], we applied the following criteria:

a) Soil samples for ^137^Cs measurements should be taken from the areas where (according to the information available from local residents and officials) no human activity was carried out, such as undisturbed areas in ancient cemeteries, edges of mature forests, unplowed virgin soil on flat surfaces.

b) The results of measurements obtained from soil samples taken near buildings or other domestic structures, as well as in ravines, ditches or on high hills should be excluded from analysis.

c) During the years after radioactive contamination, soil samples should not be superficial, but should be taken to a greater depth—at least 30 cm or more, and samples should be divided into layers 1–5 cm thick for subsequent measurements, as far as an analysis of activity distribution over depth makes it possible to assess the presence of human intervention, migration of radionuclides over depth and, most importantly, to assess the total inventory of measured activity.

But even if the above-listed criteria are met, the influence of such natural phenomena as weathering, washing out etc. cannot be excluded. Therefore, in our external dose calculations, we included only the results of sample measurements that met the three criteria, and from these samples we selected those that corresponded to the 90th-percentile of the statistical distribution of the results of their measurements. If the number of measurements was not sufficient for statistical analysis, then the measurements’ maximum values were used. [24, 31, 38, 39]

### Methodologies of retrospective absorbed external exposure dose estimation in residents of the affected settlement

#### ESR method of individual cumulative dose reconstruction using human tooth enamel

For instrumental individual external dose reconstruction an ESR method was used. A detailed description of the method, including the results of international intercomparisons and methodology for estimation of dose uncertainties has been published [18–22, 40–47]. Tooth enamel was mechanically separated from dentine using hard alloy dental drills and diamond saws. Dentins were removed carefully with cooling water to prevent the sample from heating, which can induce an additional ESR signal and significantly change the shape of the signal [18]. Tooth enamel was crushed with cutting pliers to chips 0.5–1.5 mm in diameter; two samples were prepared from the buccal and lingual parts of each tooth; the measurements were carried out in the X-band on the ESR spectrometer JEOL JES-FA100 at a stabilized room temperature of 21°C; the spectrometer was equipped with a high Q-factor cylindrical TE011 cavity model ES-UCX2, and the selected spectrum recording parameters were the same as previous studies [40–42]. Special computer software was used for spectra processing and dose estimation [43, 44]. Excess doses were calculated with accounting for tooth enamel age (year of enamel formation), and background dose [19–22]. Uncertainty of dose estimation was determined based on a semi-empirical formula published in [20].

It is important to note that the selection of ESR measurement results was accompanied by analysis special documentation related to the tooth enamel sampling in individuals and exact indentification of the pationets. This documentation is mentioned below as ‘ESR questionnaires’ or as ‘ESR documentation.’ If these ESR documents were completed, then we included these people in the intercomparison, but if these documents were not available the correponding persons were not included to the intercomparison.

#### Methodology of individual doses calculation

Calculation of individual absorbed external exposure dose to the inhabitants of study settlements was performed according to the previously-used methodology [27, 28]. When calculating individual doses, the following parameters were taken into account: an age-dependent conversion factors (from settlement-average absorbed dose to air to absorbed dose in body) depending on age of a person at the time of exposure; number of hours spent outdoors per day (this parameter depends on age and ethnicity); shielding factor, accounting for decrease of exposure rate indoor comparing to outdoor (the values of this factor depend upon the house building materials); coefficient accounting for a part time residence of an individual in the study settlement (if applicable).

The equation to assess individual external dose D_ext_ from a nuclear test affected the study settlement, for the residents of different ages, was as follows [27, 28]:


(1) \begin{equation*} {\mathrm{D}}_{\mathrm{ext}}={\mathrm{D}}_{\mathrm{air}}\times \mathrm{CF}\times \left[{\mathrm{t}}_{\mathrm{out}}+\left(24\hbox{--} {\mathrm{t}}_{\mathrm{out}}\right)/{\mathrm{k}}_{\mathrm{shield}}\right]/24 \end{equation*}

where,

D_air_—settlement-average dose to air, Gy (calculated using the approach described in [27, 28] and main parameters from Table 1).

CF—age-depended conversion coefficient from settlement-average dose to air to whole-body dose for individuals of different ages, Gy × Gy-1 (Table 2).

**Table 2:** Typical age-depended conversion coefficients from settlement-average dose to air to whole-body dose for individuals of different ages [48]

Age	CF, conversion coefficient (Gy per Gy)
3 mo	0.95
1 y	0.91
5 y	0.88
10 y	0.83
15 y	0.81
Adult	0.79

t_out_—number of hours spent outdoors per day depending on age and ethnicity (Table 3).

**Table 3:** Typical number of hours spent outdoors a day, t_out_, [33, 49]

Age	t_out_ (h)	
	Kazakhs	Russians
3 mo	1	0.5
1 y	5	9
5 y	7	9
10 y	10 (5)^a^	10 (6)^a^
15 y	13 (5)^a^	11 (3)^a^
Adult	16	16

^a^Number of hours spent outdoors a day during a school year (September 1 to May 31) is given in parentheses.

k_shield_—shielding factor, accounting for decrease of exposure rate indoor comparing to outdoor.

Typically, the house construction materials were different on the right and left banks of Irtysh River [5, 47]. In the study period, in the settlements located on the right bank of Irtysh River, all houses, with few exceptions, were wooden, whereas the houses in the settlements on the left bank were adobe. This is due to the nature specifics of the region, the right bank is occupied by pine forests, while the left bank—by steppe. Thus, it seems reasonable to attribute a single shielding factor (as a typical parameter) to each resident of all settlements located on the same bank of Irtysh River: 3—for the right bank (wooden), 13—for the left bank (adobe), and 10—for one-storied brick buildings, for example, schools and administrative buildings [33].

Analysis of available residential histories of people from the study settlements revealed that some individuals became residents in these villages after 1950, i.e. too late to receive any significant external exposure from the radioactive fallout following the nuclear test on August 29, 1949. These residents were, therefore, excluded from a comparison of ESR-based doses with calculated doses.

#### Selecting individuals with available ESR dosimetry data for the comparison of individual external ESR-based and calculated doses

To compare individual external exposure doses based on personal ESR dosimetry data with calculated individual external exposure doses, we selected the residents with ESR dosimetry results who met the following eligibility criteria: the date of tooth enamel formation had to be before 1949; a person resided in one of the study villages in the first year after the test on 29 August 1949; a person has a special ESR individual questionnaire along with tooth enamel measurements. For this purpose, we used the databases at A. Tsyb Medical Radiological Research Center—Branch of the National Medical Research Radiological Centre of the Ministry of Health of the Russian Federation (MRRC) [8, 14, 19, 21], L.N. Gumilyov Eurasian National University (ENU), Astana, the Republic of Kazakhstan [20, 22, 40, 41], and Research Institute of Radiation Medicine and Ecology (NIIRME), Semey, the Republic of Kazakhstan, [5, 47]. In all, 16 eligible persons from the study settlements were found: 10 from Dolon, four from Mostik, and two from Cheremushki. Out of 16 selected persons, seven had detailed information on residence history and lifestyle. They were considered separately in the dose calculations and comparison.

## RESULTS

### Comparison of settlement-average external doses to air

The calculated settlement-average external cumulative doses to air were compared with the results based on available LRD data and with the results of dose reconstruction based on the available measurements of ^137^Cs soil contamination density in the study settlements (Table 4). A comparison of the calculated settlement-average external doses to air with the dose LRD-based estimates and the dose estimates based on ^137^Cs soil contamination density data shows relatively acceptable agreement, considering the fundamental difference between different retrospective dosimetry methods used. Good agreement between LRD-based doses and calculated doses shows a realism of the model used to calculate doses to air based on available historical information on measured dose rates to air.

**Table 4:** Comparison of settlements-average external cumulative dose to air estimated by different methods.

	Dose estimations based on calculations, D_calc_		Dose estimations based on LRD measurements, D_LRD_		Dose estimations based on ^137^Cs soil contamination data, D_CS-137_		
Settlement	D_calc_ (mGy)	Ref. to parameters for calculations of external dose to air	D_LRD_ (mGy) recalculated to external dose to air	Ref. to conditions of sampling	D_Cs-137_ (mGy)	Ref. to ^137^Cs soil contamination data	
Dolon	450^a^ 466 ± 30^b^ 500 [28]	[27, 28, 34–37]	408 ± 30^c,d^	[3, 16]	500 [24]	[31, 38, 39]	
			498 ± 90^c,d^				
			420 ± 120^c,d^				
			475 ± 110 [9]	[9]			
Mostik	120		N/A^e^		110		
Cheremushki	200 140		N/A^e^				290

^a^Recalculated from the dose in centerline of radioactive trace [27, 34–36] to the dose in the settlement in accordance with the gradient of dose rate [37] and with gradient of radioactive contamination [12] in the direction from the centerline of radioactive trace into the village of Dolon, which is close to the radioactive trace.

^b^Calculated and averaged for locations near the four sampling points of quartz containing bricks for LRD [3, 16] in accordance with the gradient of dose rate [37] and with accounting for gradient of radioactive contamination [12] in the direction from the centerline of radioactive trace into the village of Dolon.

^c^Measured doses in bricks [10, 13, 15] were averaged over four samples of bricks sampled inside the village of Dolon [3, 16].

^d^Dose to air was estimated on the base of measured doses in the four samples of bricks using the value of MC calculated conversion factor from the dose at 10 mm depth in the bricks to the dose to air; the value of this conversion factor is equal to 2 ± 0.25.

^e^N/A - Not available.

### Comparison of individual external doses

Table 5 shows the results of comparison between external dose estimate based on ESR individual dosimetry method by human tooth enamel and calculated dose.

**Table 5:** Comparison of calculated individual external dose from the fallout for selected persons with available individual ESR measurements of tooth enamel supported by available information from personal interviews

#	Settlement	Age (age group)	Ethnicity	Calculated individual dose (mGy)			Dose estimated by ESR dose reconstruction method	
				Typical input data	Based on individual information		Year of enamel formation	Dose ±2SD^^*^^*^^ (mGy)
					from NIIRME	from MRRC		
1	Dolon	20 (adult)	Russian	264	N/A^^*^^	N/A	1934	200 ± 60
2	Dolon	18 (adult)	Russian	132	127	113	1937	54 ± 20
3	Dolon	13 (15 y)	Kazakh	19	N/A	N/A	1947	72 ± 28
4	Dolon	11 (10 y)	Kazakh	196	224	198	1947	280 ± 50
5	Dolon	25 (adult)	Russian	264	N/A	N/A	1929	350 ± 60
6	Dolon	8 (10 y)	Kazakh	196	N/A	N/A	1948	76 ± 27
7	Dolon	21 (adult)	Russian	264	264	226	1934	430 ± 90
8	Dolon	9 (10 y)	Kazakh	196	N/A	N/A	1947	143 ± 39
9	Dolon	19 (adult)	Russian	264	N/A	N/A	1933	440 ± 110
10	Dolon	6 (5 y)	Kazakh	200	N/A	N/A	1949	87 ± 33
11	Mostik	20 (adult)	Russian	111	N/A	71	1934	50 ± 30
12	Mostik	10 (10 y)	Russian	84	N/A	N/A	1944	66 ± 19
13	Mostik	18 (adult)	Russian	111	N/A	N/A	1936	92 ± 35
14	Mostik	10 (10 y)	Kazakh	83	N/A	75	1943	60 ± 44
15	Cheremushki/ Mostik	10 (10 y)	Kazakh	117	N/A	120	1945	54 ± 37
16	Cheremushki	12 (15 y)	Russian	115	N/A	121	1943	102 ± 39

^^*^^N/A – not available; ^^*^^*^^SD – standard deviation

Comments on individual external dose calculations (Table 5) are as follows.

Person #2: According to the individual questionnaire this person left the village of residence for 2 days at the time of testing. This was taken into account assessing his/her individual dose from external exposure.

Person #3: According to the data from NIIRME register, this person attended school in Dolon but lived in another village. For the calculation of external dose using typical input data, it was assumed that he/she was not in Dolon until September 1, 1949. From this date it was assumed that he/ she spent 6 hours/day indoors the school (wooden building, k_shield_ = 3) and 1 hour/day outdoors in Dolon.

Person #4: According to the individual questionnaire, this person spent 15 hours/day outdoors, but according to additional information from the NIIRME register, the person (female) was at school at the time of testing. For dose calculation using the typical data set it was assumed that she was a schoolgirl. For dose calculations using the questionnaire she spent 15 hours/day outdoors until September 1 and 5 hours/day outdoors as a schoolgirl from that date on. These values were used for the corresponding individual dose calculation based on NIIRME data. In the questionnaire obtained from MRRC it was written that she spent in average about 8 hours/day outdoors. This value was used for the corresponding individual dose calculation based on MRRC data.

Person #7: According to the individual questionnaire from NIIRME, this person spent 8 to 16 hours/day outdoors. According to the individual questionnaire from MRRC, the person spent 8 to 12 hours/day outdoors. These values were used for the corresponding individual dose calculation based on NIIRME and MRRC data.

Person #15: According to the individual questionnaire, this person lived in Cheremushki until January 1, 1950 and then moved to Mostik.

**Fig. 2:**
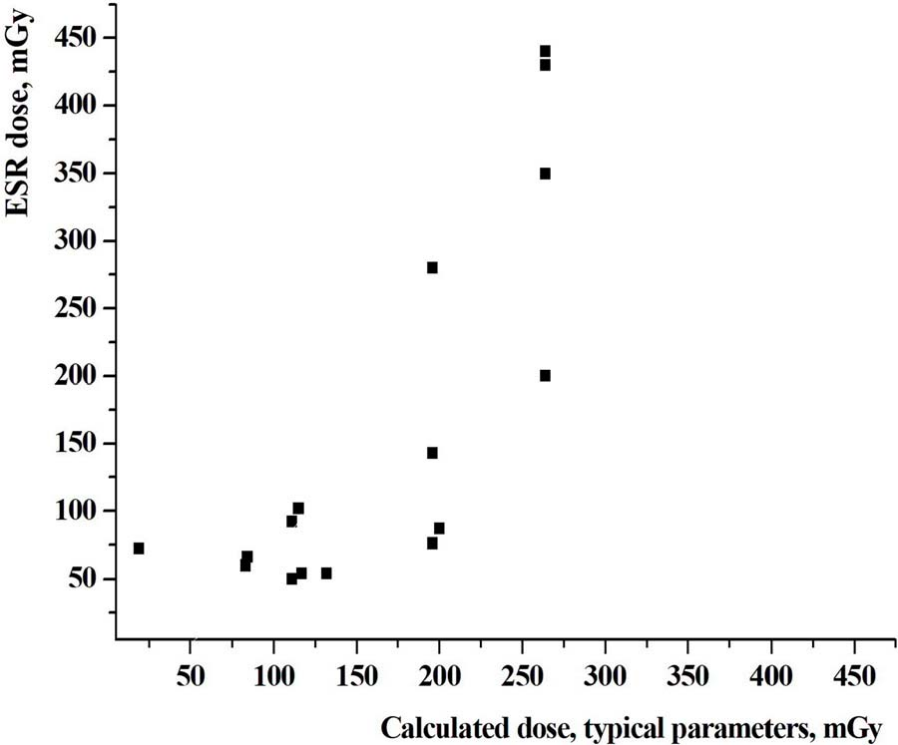
A comparison of calculated cumulated external dose estimates based on typical input data with the dose estimates based on ESR measurements of tooth enamel (ESR dose) in the same people (*n* = 16 persons).

A comparison between values of calculated cumulative external dose estimates based on typical input data with individual dose estimates based on the ESR measurements is shown in Fig. 2.

**Fig. 3:**
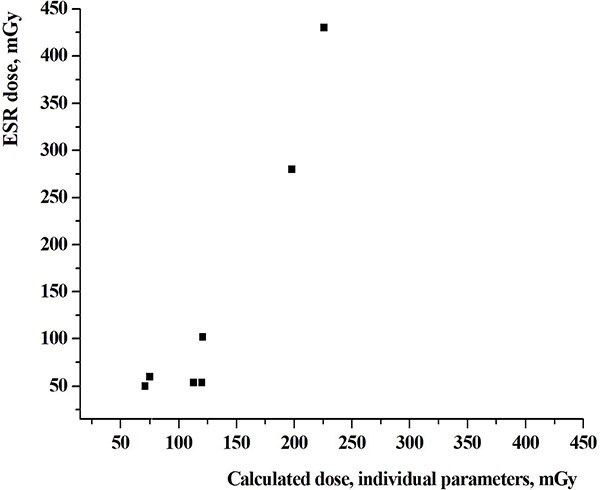
A comparison of calculated cumulated external dose based on individual information from questionnaires with the dose estimates based on ESR measurements of tooth enamel (ESR dose) in the same people (*n* = 7 persons).

A comparison of calculated cumulative external dose estimated on the base of individual information from the questionnaires with dose estimates based on ESR measurements is shown in Fig. 3.

**Fig. 4:**
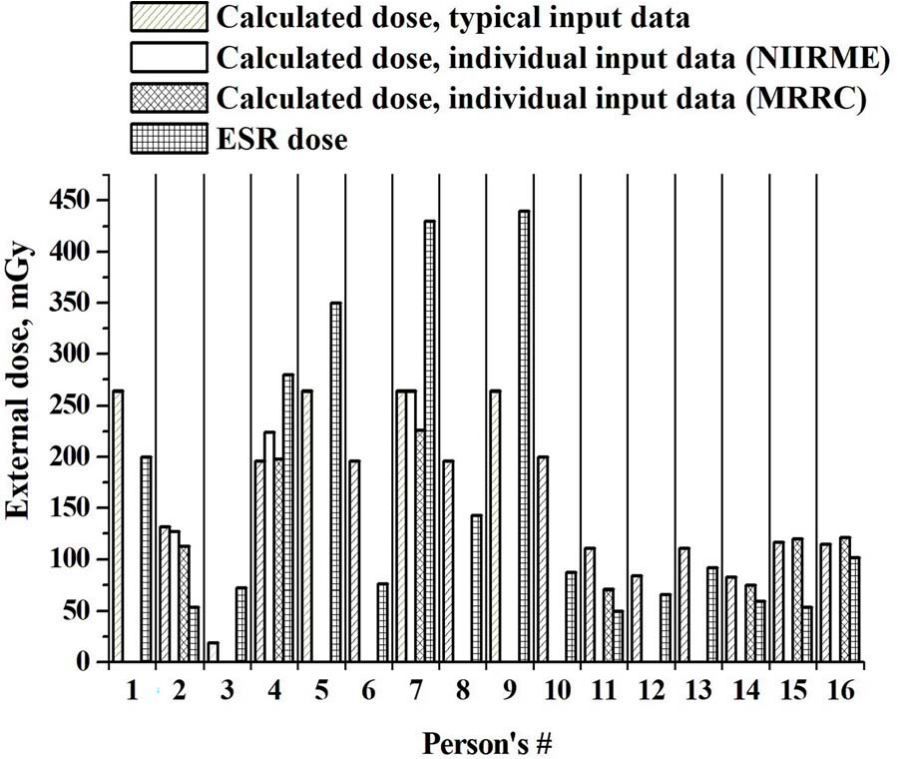
A comparison of individual external doses estimated using various data sources and different methods of retrospective dosimetry.

Correlation coefficients between the two sets of doses shown on Fig. 2 and Fig. 3, were 0.782 and 0.940, respectively. The results of comparison of individual external doses estimated using various data sources and different methods of retrospective dosimetry for all 16 persons showed on Fig. 4.

Doses calculated using data from the individual questionnaires had a better correlation with ESR-based doses than with the doses calculated using typical input data. This is because personal questionnaires provide more detailed information about individual behavior.

## DISCUSSION

As it was shown for Dolon village, the LRD method with quartz inclusions in bricks sampled from the settlement is useful for validation of the calculated average cumulated external absorbed dose to air. But it should be noted that the possibilities of collecting quartz-containing samples from buildings that were ‘witnesses’ of time-remote events of radiation exposure are very limited for the Semipalatinsk region [9, 16], as there were a few brick buildings in those years [10–13, 15].

Therefore, in a case of absence of archival data with the results of dose rate measurements and LRD dose data, reconstruction of external exposure can be supplemented with the data from later measurements of soil contamination with long-lived radionuclides, such as, ^137^Cs. However, it is to note that according to [23], dose estimates based on ^137^Cs soil contamination data can be considered as a surrogate only, as far as these estimates can be overestimated if soil sampling sites are located far from radioactive trace. It was not related to the study settlements because all the settlements were located quite close to the trace (Fig. 1).

A comparison of calculated individual cumulative external dose estimates for people from Dolon, Mostik and Cheremushki villages using typical input data with individual dose estimates performed by ESR retrospective dosimetry method in the same people showed good correlation between the two sets of dose estimates with a correlation coefficient of 0.782.

A comparison of calculated individual cumulative external dose estimates performed for seven persons using individual interview data with cumulative individual dose estimates performed by ESR dosimetry method in the same people, showed better correlation between the two sets of dose estimates with a correlation coefficient of 0.940.

Here it is to note that there was an indication on medical exposure in the corresponding documentation of teeth enamel sampling for ESR measurements in two persons (persons #7 and #9 in Table 5). The documents were filled up at local hospitals by medical personnel. Unfortunately, data on medical radiation doses and corresponding explanations about type of medical exposure were not available. According to information from local medical authorities this could be a chest X-ray screening of the lungs which is commonly used medical procedure in local hospitals for diagnosis of tuberculosis etc. Usually, this procedure is carried out not more than one time per year. For such examination, typical radiation dose is less than 0.5 mSv per procedure [50, 51]. That is, over 70 years the accumulated dose will be <35 mSv, which is essentially less in comparison with the values of calculated doses and with the values of measured ESR doses in these two persons. According to their anamneses, these two persons were not subjected to radiation therapy or dental radiography. In this regard, the contribution of medical exposure to the results of ESR dosimetry was considered only as possible, but not certain, and these two individuals were included in the total number of individuals to compare calculated and measured doses. However, the impression remains that these two individuals had an additional unknown exposure, which led to the elevated ESR dose measurements. Unfortunately, their life histories are not available in full, precluding from a precise conclusion about the possible source of this exposure.

In general, calculated cumulated doses using data from individual questionnaires correlate better with cumulated ESR-based doses in the same people, than the doses calculated using typical input data. This is because the data from individual questionnaires provide more detailed information on relocation and on the number of hours spent outdoors by a specific person.

The sample size used for the comparison exercise was relatively small, only 16 people. It is important to continue this work to compare dose estimates for a larger sample that includes people with available individual questionnaires and ESR measurement results.

Overall, the comparison of external dose values estimated using different retrospective dosimetry methods in the three settlements located nearby the SNTS showed a reliability of the calculation method used for the retrospective assessment of settlement-averaged external doses to air and individual external doses for the residents. This is important for planning and performing radiation-epidemiological studies in this region to assess potential impact of low doses radiation exposure on peoples’ health.

## CONFLICT OF INTEREST

The authors declare no conflict of interest.

## AUTHOR CONTRIBUTIONS

Conceptualization, V.S., S.Sh., M.H., K.A.; methodology, V.S., S.Sh., A.L.; validation, E.O., B.G., P.Sh.; formal analysis, A.Ka., S.I., P.Sh., NF, NK; investigation, V.S., S.Sh., K.Zh., A.L., V.B., V.Ia.; resources, A.Ka., S.I., P.Sh., K.A.; data curation, M.Ya., A.S., S.E., Z.A., A.A.; writing—original draft preparation, V.S., S.Sh.; writing—review and editing, E.O., A.Ke.; supervision, M.H., A.Ka., S.I., P.Sh., K.A.; project administration, M.H., A.Ka., K.A., A.Ke.; funding acquisition, M.H., A.Ke. All authors have read and agreed to the published version of the manuscript.

## FUNDING

The research was supported by Japan Society for the Promotion of Science (KAKENHI) grant (grant 19H01149) to Professor Masaharu Hoshi, Member of JRRS, by Environmental Radioactivity Research Network Center (ERAN) (F-22-14 and F-23-14), and by grant of Ministry of Science and Higher Education of the Republic of Kazakhstan for scientific and scientific and technical projects (grant № AP19678341); supported by the A. Tsyb Medical Radiological Research Centre – branch of the National Medical Research Radiological Centre of the Ministry of Health of the Russian Federation in a framework of of bilateral International Agreements on the scientifical cooperation with Hiroshima University (Japan), Research Institute of Radiation Medicine and Ecology (Republic of Kazakhstan), and L.N. Gumilyov Eurasian National University (Republic of Kazakhstan).

## ETHICAL APPROVAL

Ethical approval from the Ethics Committee of Scientific Research Institute of Radiation Medicine and Ecology of the Semey Medical University, Semey, Republic of Kazakhstan, was obtained prior to conducting this study (Document No 3, 23.06.2018).

## INFORMED CONSENT STATEMENT

Informed consent was obtained from all subjects involved in the study: all subjects were informed why this research is being conducted, about assuring of the anonymity, how their data will be used and if there are any risks associated.

## DISCLAIMER

Where authors are identified as personnel of the International Agency for Research on Cancer/World Health Organization, the authors alone are responsible for the views expressed in this article and they do not necessarily represent the decisions, policy or views of the International Agency for Research on Cancer/World Health Organization.

## DATA AVAILABILITY

All authors are ready to provide data upon request.
